# Preliminary assessment of the leukocyte coping capacity as a point of care marker in horses with stress associated diseases

**DOI:** 10.1186/s12917-025-05179-9

**Published:** 2025-12-07

**Authors:** Vendula Jandová, Nikolaus Huber, Fatma Graiban AlMheiri, Karolína Bábor, Dagmar S. Trachsel

**Affiliations:** 1 Equine Internal Medicine Practice, Svinčany 165, Czechia, 535 01 Czech Republic; 2 Clinical Department for Farm Animals and Food System Science, Center for Food Science and Veterinary Public Health, University of Veterinary Medicine Vienna, Veterinärplatz 1, Vienna, 1210 Austria; 3 Oxford MediStress Ltd, 267 Banbury Rd, Summertown, Oxford, OX2 7HT UK; 4 Central Veterinary Research Laboratory, P.O. Box 597, Dubai, UAE; 5 MVDr. Nebovidská 69, Střelice, 664 47 Czech Republic; 6 Clinical Department for Small Animals and Horses, Clinical Centre for Equine Health and Research, University of Veterinary Medicine, Veterinärplatz 1, Vienna, 1210 Austria

**Keywords:** Allostatic load, Gastric ulcer severity score, Neutrophil oxidative burst, PMNL, Polymorphonuclear cells, Equine

## Abstract

**Background:**

Stress represents a serious health and welfare concern; however, its objective assessment remains difficult. The equine gastric ulcer syndrome (EGUS) and orthopedic diseases that cause pain are among stress associated diseases in equine medicine. The leukocyte coping capacity (LCC) quantifies oxygen radical generation of neutrophil granulocytes which is altered under stress. Therefore, LCC could be a novel biomarker for stress in horses and we hypothesized that horses with stress associated diseases would have lower LCC values in comparison to horses without these diseases.

**Materials and methods:**

In this observational clinical pilot study, 45 privately owned horses were classified according to the most relevant clinical diagnosis based on clinical, laboratory and gastroscopic findings into the following groups: (1) *No EGUS*; no clinical and/or laboratory and/or gastroscopic signs of EGUS, lameness or other diseases, (2) *EGUS*; any grade of EGUS, but no clinical and/or laboratory signs of lameness or other diseases, (3) *Lameness*; any grad of lameness, but no clinical and/or laboratory signs other diseases, any grade of EGUS possible, (4) *Other diseases*; identified based on abnormal findings in clinical examination and/or laboratory work, with no evidence of lameness, any grade of EGUS possible. The LCC was measured at first visit (T1) and 28 days later (T2) and the values compared among the groups with mean comparison tests and mixed effect models for repeated measures.

**Results:**

Primary results indicate that horses in group 3 had significantly (*P* = 0.012) lower values for LCC compared to horses in the group 1 at T1. Also group 3 horses had highest EGUS scores. At T2 LCC was still significantly lower in this group (*P* = 0.031), even though the severity of EGUS decreased in all horses with treatment (*P* = 0.004).

**Conclusion:**

Lame horses had higher EGUS scores and lower LCC levels, indicating a possible link between lameness, EGUS, and stress. Our findings support further investigation into the use of LCC as a quantitative immunological marker of stress with strong potential for use at point of care.

**Supplementary Information:**

The online version contains supplementary material available at 10.1186/s12917-025-05179-9.

## Background

Stress and stress-related diseases are a considerable welfare issue in horses and domestic animals in general. However, despite extensive research efforts the objective assessment of stress remains difficult [1–3]. Hormone levels of the Hypothalamic-Pituitary-Adrenal (HPA) axis have largely been used to assess stress levels [4–10]. Further, physiological parameters, such as changes in the heart rate variability (HRV) or behavioral scores/ethograms have also been applied in this context [3, 5–9, 11–14].

Stress is defined as a challenge to homeostasis, which is perceived as such and provokes a stress response in the body. The ensuing physiological changes in body systems, as well as the number and intensity of these dynamic changes (i.e., allostatic load), define the pathophysiological costs of stress [15–18]. Duration, timing and severity of the stress response are central in this reaction [15–17], whereas long-term stress (i.e. hours to days) is believed to have more pronounced and negative health consequences compared to short-term stress (i.e. minutes) challenges [17, 18].

Stress, or pain, as a strong stress trigger, can significantly affect the function of innate immunity cells. Neutrophil granulocytes (NEU) are the first line of defense in the innate immune system. Stress or pain reduces NEU capacity to produce and release reactive oxygen species (ROS), thereby weakening this defense mechanism [19–21]. The leukocyte coping capacity (LCC) quantifies the ability of leukocytes (LC), and in particular, NEU, to produce ROS in response to activation [21–23]. In veterinary medicine, LCC has been mainly used to quantitatively assess stress in the context of wildlife manipulations and short- to medium-term stress [19, 20, 24, 25]. For example, in wild equids, LCC was significantly reduced in individuals that showed obvious signs of stress during transport [19]. Beyond capture and physical restraint, LCC may also be affected by longer term stress with a psychological component as shown for water voles and non-human primates in different housing systems [26, 27] or for bears and water voles in difference social groups [25, 28]. LCC measurements were lower in non-human primates maintained in cages compared those housed in open-room systems [26]. Similarly, water voles housed in laboratory cages showed lower LCC measurements than those housed in outdoor systems [27] and solitary bears had lower LCC measurements than those in family groups [25]. Water voles held in larger groups had lower LCC measurements than those held in smaller groups, implying more severe stress in larger groups [28]. In human studies, LCC has been used as a marker to objectivize psychological or disease related stress [29–31]. In domesticated animals LCC has been used to assess pain in calves and stallions after castration [32, 33], and heat stress in growing pigs [34], but there are few publications of LCC as a stress marker in domesticated healthy or diseased horses [35].

In equine medicine, a disease that is commonly associated with management factors leading to stress is the equine gastric ulcer syndrome (EGUS), which encompasses equine squamous gastric disease (ESGD) and equine glandular gastric disease (EGGD) [36]. Over time, it has become apparent that these are two different diseases primarily due to major differences in their etiopathogeneses. ESGD is caused by prolonged contact with acidic content of the stomach [36, 37] and can be favored by several management factors [38, 39] and high-intensity training [40–42] that lead to a high-stress load and contribute to an increase in the exposure time of the squamous mucosa to gastric acids. In contrast, the pathophysiology, though not entirely elucidated for EGGD, indicate that possible drivers of EGGD include impaired protective mechanisms of the glandular mucosa or long-term stress associated with higher stress sensitivity [43–45]. Thus, even though the pathophysiology is complex and the etiological relationship between the two forms of EGUS has yet to be clarified, it seems likely that, even though not the primary driver, a state of stress is involved in the development of both forms of EGUS [36, 43]. Another source of stress, orthopedic diseases, commonly associated with lameness, gait abnormalities or altered behavior, can be assessed by ethograms or pain scores [14, 46]. The alleviation of the behavioral changes and gait abnormalities by local analgesia clearly associate these changes with reduction in pain, discomfort and ensuing stress [13, 47]. Other acute or chronic diseases in horses have also been shown to represent a challenge for the organism leading to increased stress levels with higher cortisol and ACTH levels and relevant changes in other biomarkers of stress, such as serotonin, adrenaline or noradrenaline [10].

The aim of the study was to explore the value of LCC measurements to quantitatively assess stress in a disease context in horses. Therefore, we measured LCC levels in horses presented primarily for gastroscopy in a first line internal medicine ambulatory private practice to assess whether the presence of EGUS would be associated with lower LCC levels. However, some horses presented for gastroscopy have concurrent other conditions leading to stress, for example orthopedic diseases that might or might not have been noticed by the owners [48–50]. Therefore, our hypothesis was that that horses with diseases leading to (e.g. orthopedic diseases) or caused by stress (e.g. EGUS), will show decreased LCC levels indicating a higher stress load compared to horses that would show no signs of pain or disease (e.g. neither lameness nor gastric ulcers).

## Materials and methods

### Study population

**Fig. 1:**
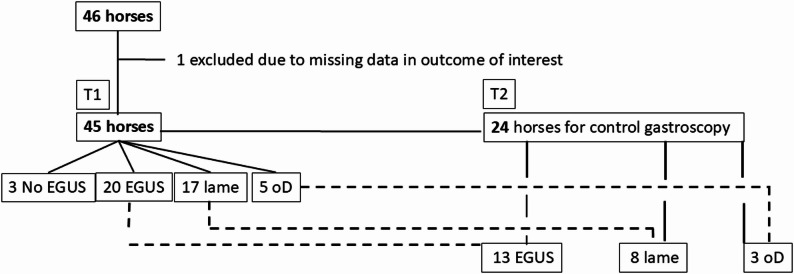
Study population available at first (T1) and second visit (T2). The groups are defined as described in materials and methods. Of the 20 horses in *EGUS* group only 13 were available at T2, of the 17 horses in the *Lame* group only 8 were available at T2 and of the *oD* group only 3 were available on T2. EGUS, Equine Gastric Ulcer Syndrome; oD, other diseases

In this observational clinical pilot study running between April 2023 and September 2023 horses presented for gastroscopy to a first line internal medicine ambulatory private practice in the Czech Republic either due to gastrointestinal signs suggestive of gastric ulcers or at the owner’s request were included in the study (see Fig. 1).

All procedures were clinically indicated, performed in accordance with good veterinary practice and local legislation. No specific veterinary treatments or interventions were carried out solely for the purpose of this study. Prior to inclusion in the study, informed consent was obtained from the owner.

### Study protocol

At first visit (T1) a complete clinical examination of each horse was performed including assessing gait by trotting the horses in hand. The overall lameness score was graded as 0 no lameness to 5 + non-weightbearing (AAEP Lameness scale) [51]. For each horse, the summary of the history and clinical examination as well as the breed, sex, body weight (BW, using horse weight tape, Divine Animals, s.r.o. Křižany, Czech Republic, or a livestock weight scale CAS VT 1500), body condition score (BCS) [52], inner body temperature (IBT), heart rate (HR) and respiratory rate (RR) were recorded.

Blood was collected for the hematological (EDTA tubes) and biochemical (lithium heparin tubes) analyses by punction of the jugular vein after application of antiseptic and prior to administration of sedation (detomidine hydrochloride, 0.01–0.02 mg/kg BW (Detonervin 10 mg/ml, Sevaron) combined with butorphanol, 0.01 mg/kg BW (Torphadine 10 mg/ml, Sevaron), for the gastroscopy. The remainder of the blood collected was used for LCC measurement. The gastroscopies (Gastroscope by Karl Storz, Typ TELE PACK VET X LED, length 300 cm, diameter 10.4 mm) were performed in the home stables as described by Sykes and Jokosalo [53].

All gastroscopies were performed, and findings graded by two European College of Equine Internal medicine (ECEIM) specialists. In the squamous part of the stomach, the ulcers were graded from 0 to 4 according to the ECEIM consensus statement [36]. The glandular ulcers were described according to the ECEIM consensus statement [36] and further graded according to the grading system proposed by Sundra et al. [54]. In order to obtain better discrimination of the severity of the ulcers in the glandular part of the stomach, a recently published scoring system was also applied [55]. In this scoring system the severity was assessed by giving points to lesions as described in Table 1. A total severity score (TSS) was obtained by adding the numeric value of the ESGD score and the numeric value of the score as defined in Table 1. Overall grading of EGUS is detailed in Appendix A supplementary material Table S3.

**Table 1:** Additional scoring system for equine glandular gastric diseases according to Scott et al. 2023 [55].

	Presence of Fibrin (F)	Presence of Hemorrhage (H)	Presence of Erythema (E)	Estimation of Lesion Area (A)	
Not observed	0	0	0	Small	1
Mild	1	1			
Moderate	2	2	1	Medium	2
Severe	3	3		Large	3
Total Score	= (Score F + Score H + Score E) x score A				

According to the findings, treatment with omeprazole (commercially available buffered formulation at a dosage of 4 mg/kg po SID) and/or sucralfate (compounded formulation at a dosage of 12 mg/kg po BID) was offered to the owners based on the clinician assessment. A follow up gastroscopy to be carried out during a second visit (T2) 28 days later was recommended to the owners. Causes, pathophysiology and management strategies of gastric ulcers were discussed with the owners, but compliance with the advised change in management or use could not be verified. Further diagnostic tests for all relevant findings were proposed to the owners, but it was up to them whether, when or by which veterinarian these further investigations were performed.

If the owner presented the horse for a follow up visit (T2), complete clinical examination, hematological analyses and gastroscopy was performed in similar fashion as at T1.

Inclusion criteria for T1 were (i) gastrointestinal signs suggestive of gastric ulcers reported by the owners or (ii) the gastroscopy was requested by the owners and (iii) an informed consent was given by the owners. Excluded were uncooperative horses that made a gastroscopic examination impossible even under sedation and horses for which it was not possible to complete the blood analyses (for reasons such as insufficient blood sampling volumes, technical problems during analyses etc.). The number of included horses at T1 and for a second sampling at T2 are shown in Fig. 1.

Full hematological and biochemical analyses were performed in order to rule out concurrent diseases as differential diagnosis for the clinical sings described in the history. The results of the blood work were also used for disease group allocation in the further analysis. The hematological analyses (including a complete white blood count with differential and a complete red blood count including hematocrit, hemoglobin, and platelet count, as shown in Appendix B supplementary material Figure S1-S6) were performed at the end of the sampling day with automatized cell counters at two sites depending on which laboratory was nearer to the stables (Automated Hematology Analyzer Mindray BC-6200 or a fully automatic cell counter, Exigo H400, LABtechnik, s.r.o, Brno).

The lithium heparin plasma was collected after centrifugation as soon as possible post sampling and stored at −20 °C until analyzed. The biochemistry analyses included total protein (TP), albumin (ALB), globulin (GLOB), alkaline phosphatase (ALKP), aspartate-aminotransferase (AST), gamma-glutamyl-transferase (GGT), total bilirubin (TBIL) blood urea nitrogen (BUN), creatinine (CREA), creatine kinase (CK), lactate dehydrogenase (LDH), glucose (GLU) and calcium (Ca) (IDEXX Catalyst One, IDEXX Vet Med Labor GmbH, Vienna).

### Disease group allocation

For the statistical analyses, the horses were classified into one of the following groups according to the most relevant clinical diagnosis based on clinical, laboratory and gastroscopic findings at T1 as follows:

−1) No EGUS, no abnormal findings on clinical examination, gait examination (grade 0/5) or blood work, were identified. Gastroscopy revealed no ulceration in the squamous (grade 0/4) or glandular parts (grade 0/4) of the stomach.
−2) EGUS, no abnormal findings on clinical examination, gait examination (grade 0/5) and blood work, were identified. Gastroscopy revealed ESGD (grade ≥ 1/4), EGGD (grade ≥ 1/4) or both and therefore the gastroscopic findings were considered to be the leading clinical diagnosis in this group.
−3) Lame, no abnormal findings on clinical examination, and blood work were identified, but the horse showed abnormality in the gait that was judged to be clinically relevant (grade ≥ 1/5). Therefore, lameness was considered to be the leading clinical diagnosis. Gastroscopy revealed either no ulceration or ESGD (grade ≥ 1/4), EGGD (grade ≥ 1/4), or both. If ulcerations were present, they were judged as concurrent to the lameness.
−4) Other diseases (oD), abnormal findings on clinical examination and/or in the laboratory work were identified leading to the primary diagnosis as shown in Appendix A supplementary material Table S2. There was no clinically relevant gait abnormality (grade < 1/5). Gastroscopy revealed no ulceration or if gastroscopy revealed ESGD (grade ≥ 1/4), EGGD (grade ≥ 1/4), or both, they were judged as concurrent to the other identified disease.

The grading of EGUS in each group was according to EGUS consensus statement and Sundra et al. [36, 54], as detailed in Appendix A, supplementary material Table S3.

All horses were examined for diagnostic purpose in an ambulatory private practice, where a clear medical indication was needed for intervention. Given this context there was no justification to perform a gastroscopy on horses that would not have had any history or without clinical signs suggestive of gastric ulcers. Therefore, it was not possible to include a group of healthy horses and the group “*No EGUS”* was, therefore, used as a reference category for this observational clinical pilot study.

### Leukocyte coping capacity

The LCC analyses were performed according to Huber et al., 2019 [19]. In brief, within 30 min after blood sampling and before knowing the findings of the gastroscopy, LCC was measured with a portable chemiluminometer (Leukocyte Coping Capacity™, Oxford MediStress Ltd.) every 5 min over 90 min under constant environmental conditions. The LCC was expressed in relative light units (RLU) [19]. The protocol used stipulated that 90 µl of 10^− 4^ mol/L luminol solution (Sigma-Aldrich, Austria, prepared by dissolution in dimethyl sulfoxide (DMSO; Merck, Darmstadt, Germany) and further dilution to the desired concentration with phosphate-buffered saline (PBS 0.01 mol/L, pH 7.4)) was added into a polystyrol antireflective tube (Greiner Bio-One, Munich, Germany). Then 10 µl of the lithium heparinized whole blood was added, and the reaction initiated with 10 µl of Phorbol Myristate Acetate (PMA) of 10^− 5^ mol/L (Merck, Darmstadt, Germany and THP Medical Products, Vienna, Austria) prepared by dissolution in DMSO (Merck, Darmstadt, Germany) and further dilution to the desired concentration with PBS 0.01 mol/L, pH 7.4). For each sample, a control tube was run in parallel where PMA was replaced by PBS 0.01 ml/L, pH 7.4. Between measurements the tubes were protected from light and incubated at 37.5 °C in a block heater (MBDB-01 Mini Dry bath, Cleaver scientific, Rugby, UK). Further, the LCC RLU values were divided by NEU count (LCC: NEU ratio) to correct for a potential mass effect.

### Statistical analyses

The descriptive data are presented as means with standard deviations (SD), medians with first and third quantiles (Q1, Q3), and as ranges including maximum (max) and minimum (min) values. For descriptive analyses due to the low number of horses in some of the groups, nonparametric analyses were used. The demographic continuous data and laboratory results were compared among the groups by the Kruskal-Wallis test with the Dunn’s multiple comparisons test or a Wilcoxon signed rank test and a Fisher’s Exact test for categorical data. A Wilcoxon signed rank test was also used to assess changes in severity score between the first and second assessments.

The LCC and LCC: NEU ratios were compared among the groups at T1 with linear mixed effect models for repeated measures while robust standard errors were included in the analyses. For the models, a random intercept for the level horses was included and an autoregressive covariance matrix was used as the values were measured from 0 min to 90 min. In these models the outcomes of interest were LCC or LCC: NEU ratio. The disease group (reference category *No EGUS*), the age and the TSS were chosen as predictors. The variables breed, sex, BW, BCS, IBT, HR or RR were not included in the models due to the low number of horses in some of the groups and because it was considered less probable that these variables would have an effect on the function of the NEU. The goodness of the models and whether age or TSS had to be included was verified based on the Akaike’s information criterion (AIC) and the Bayesian information criterion (BIC).

Further, the area under the curve (AUC) considering all measurements between 5 min and 90 min were calculated for LCC at T1 (AUC_LCC_) and LCC: NEU ratio (AUC_LCC: NEU_) at T1 and T2. The AUC were compared between disease groups using linear regression models considering age and TSS as additional predictors. Goodness of fit of the models was verified by AIC and BIC.

Additionally, the correlation between the maximal reached values of the LCC and LCC: NEU ratio curves and corresponding AUC was assessed by Spearman correlation.

For the analyses with the subpopulation that was available for follow up gastroscopy at T2 only LCC: NEU ratio was considered the outcome of interest. Due to the low number in this subpopulation LCC: NEU ratio was compared among disease groups using bivariable linear mixed effects models for repeated measurements, while initially age, treatment, severity of EGUS, whether the horses were still worked at the same level as at T1 served as potential second additional predictors. Goodness of fit of the models was verified by AIC and BIC.

For the statistical analyses GraphPad Prism software 9.5.1 (www.graphpad.com, GraphPad Software Boston, MA, USA), R Version 4.3.1 (R Core Team (2021). R: A language and environment for statistical computing. R Foundation for Statistical Computing, Vienna, Austria. https://www.R-project.org/) and SAS-Software 9.4 (SAS Institute Inc., Cary, NC, USA) were used. *P*-Values < 0.05 were considered as statistically significant.

## Results

### Initial visit (T1)

Data from 46 horses were included in the study and the classification in the disease groups, as well as the number of horses available for a follow up gastroscopy are shown in Fig. 1. The horses were used as riding horses with training intensities varying between pleasure horses and amateur sport competitions at various levels or riding school horses. One horse had to be excluded from the analyses because of technical problem during the hematological analysis and ensuing missing value for the outcome of interest LCC: NEU ratio.

The demographic description of the study population at T1 is shown in Table 2 and in Appendix A supplementary material Table S1. The lameness score in the *Lame* group was 1 + in 10 cases, 2 + in 5 cases and 3 + in 2 cases. Further, diagnostic investigation to identify lameness were left to the owner’s discretion. The diagnoses of the horses in the group *oD* are shown in Appendix A supplementary material Table S2.

**Table 2:** Demographic description of the included study population at T1

			Groups (see Material and Methods)			
	Total		No EGUS	EGUS	Lame	oD
*n*	**45**		**3**	**20**	**17**	**5**
Age (years)						
Mean (SD) median (Q1; Q3) (min; max)	11.6 (6.0) 11.0 (7.0; 17.0) (3.0; 23.0)		13.7 (5.8) 17.0 (7.0; 17.0) (7–17)	9.7 (5.0) 8.5 (5.5; 13.0) (3.0; 21.0)	14.7 (6.4) 13.0 (10.0; 20.0) (4.0; 23.0)	7.2 (3.5) 5.0 (5.0; 11.0) (4.0; 11.0)
*P* value		0.03	Ref	0.92	0.99	0.47
Sex						
g [*n*, (%)]	18 (40.0)			10 (50)	5 (29.4)	3 (60)
f [*n*, (%)]	27 (60.0)		3 (100)	10 (50)	12 (70.6)	2 (40)
*P* value		0.27				
BW (Kg)						
Mean (SD) median (Q1; Q3) (min; max)	567.4 (63.1) 574.5 (535.5; 601.0) (377.0; 720.0)		635.3 (78.1) 620.0 (566.0; 720.0) (566.0; 720.0)	553.8 (69.6) 564.5 (504.5; 594.5) (377.0; 664.0)	560.9 (43.8) 567.0 (535.5; 591.0) (465.0; 642.0)	601.6 (59.1) 605.0 (580.0; 624.0) (519.0; 680.0)
* P* value		0.16	Ref	0.26	0.28	0.99
Breed						
WB [*n*, (%)]	37 (82)		3 (100)	14 (70)	15 (88.2)	5 (100)
TBH [*n*, (%)]	2 (5)			1 (5)	1 (5.9)	
STB [*n*, (%)]	3 (7)			2 (10)	1 (5.9)	
Haflinger [*n*, (%)]	1 (2)			1 (5)		
Riding pony [*n*, (%)]	1 (2)			1 (5)		
Welsh part bred [*n*, (%)]	1 (2)			1 (5)		
*P* value		1.0				

*BW* Body weight, *EGUS* Equine Gastric Ulcer Syndrome, *f* female, *g* gelding, *n* number, *oD* other diseases, *STB* Standardbred, *THB* Thoroughbred, *WB* Warm blood

**Fig. 2:**
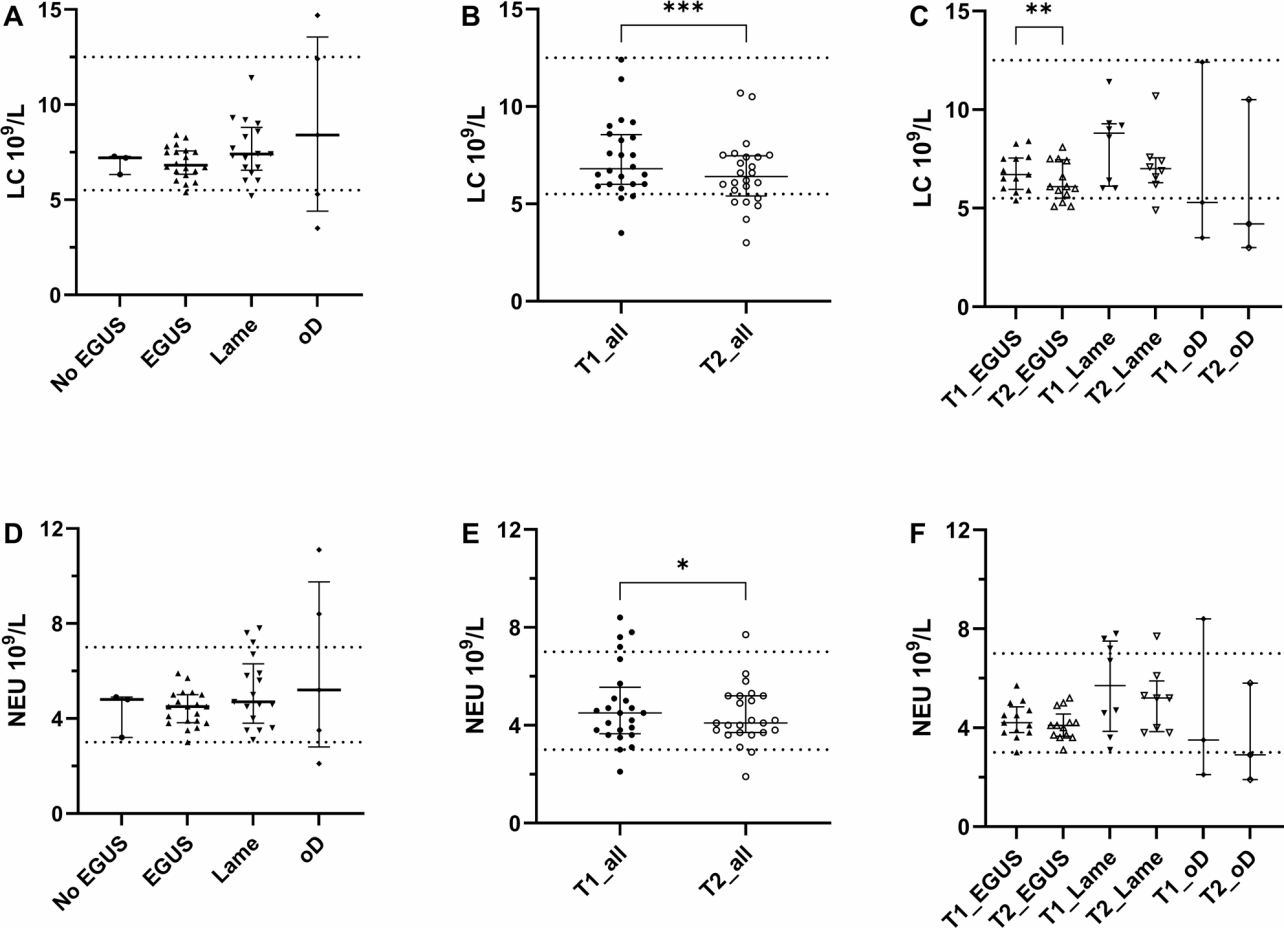
Leukocyte (LC) count (**A**) and neutrophilic granulocyte (NEU) count (**D**) at initial visit (T1) in the different disease groups (*n* = 45). Change in LC and NEU count between T1 and the second visit (T2) for the 24 horses that had a follow up gastroscopy performed (**B**, **E**), and in the different disease groups (**C**, **F**). EGUS, Equine Gastric Ulcer Syndrome; oD, other diseases; *, *p* < 0.05; **, *p* ≤ 0.01; ***, *p* ≤ 0.001. The dashes lines represent the reference values as defined by the used laboratory method

Most of the hematological and biochemical analyses were within reference ranges and there were no statistically significant differences in these parameters between the disease groups (Fig. 2 and Appendix B supplementary material Figure S1-S6). Especially in the *No EGUS* group all laboratory parameters except for the lymphocyte and monocyte numbers in one horse where within reference range. (Appendix B supplementary material Figure S1-S6). Some isolated values were outside the reference ranges but were not consistent with an organ involvement or could be explained by suboptimal storage or transport conditions prior to analysis [56–58]. The leukocyte (LC) count or the NEU count did not differ among the disease groups at T1 (Fig. 2, A and D) and reflected the disease group classification as expected. The number of LC (*p* = 0.0002) and NEU (*p* = 0.03) counts decreased significantly between T1 and T2 in all horses (Fig. 2, B and E). The LC count also decreased significantly in the *EGUS* group (*p* = 0.008, Fig. 2C).

**Fig. 3:**
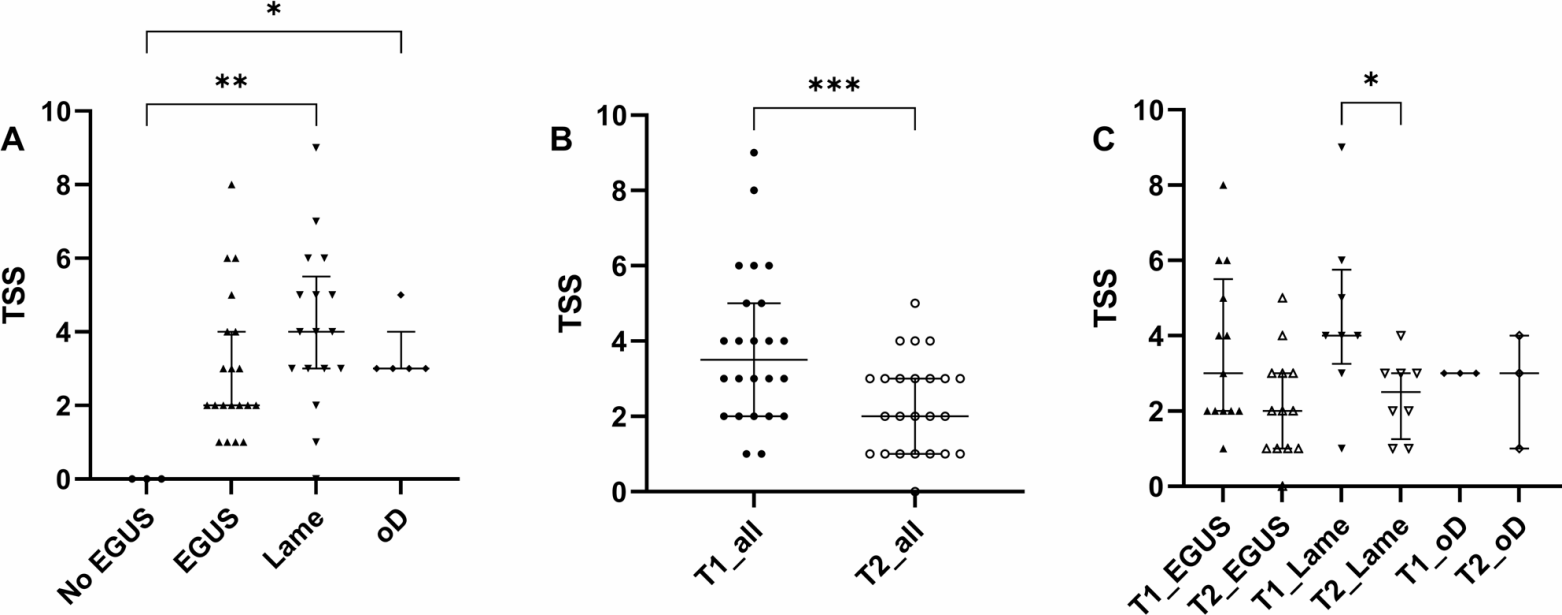
Total severity score (TSS) at initial visit (T1) in the different disease groups (*n* = 45, **A**). Change in TSS between T1 and the second visit (T2) for the 24 horses that had a follow up gastroscopy performed (**B**), and in the different disease groups (**C**). EGUS, Equine Gastric Ulcer Syndrome; oD, other diseases; *, *p* < 0.05; **, *p* ≤ 0.01; ***, *p* ≤ 0.001

The severity of gastroscopic findings and their grading and scores at T1 are presented in Table 3, in Appendix A supplementary material Table S3 and in Fig. 3. In the *Lame* group 1 horse showed no evidence of EGUS. All of the other horses in the study showed some degree of EGUS except the horses in the *No EGUS* group. The Fisher’s Exact test showed a significant difference in the distribution of the type of EGUS among the groups (*p* = 0.02). The proportion of horses that showed both forms of EGUS (ESGD and EGGD) was more often seen in the *Lame* group (12/17, 70.6%) and in the *oD* group (4/5, 80.0%). Similarly, the TSS in the *Lame* group (*p* = 0.004) and in the *oD* group (*p* = 0.04) were significantly higher than in the *No EGUS* group.

**Table 3:** Severity of the gastroscopic findings at T1

			Groups (see Material and Methods)			
	Total		No EGUS	EGUS	Lame	oD
*n*	45		3	20	17	5
Total severity Score (TSS)						
Mean (SD) median (Q1, Q3) (min, max)	3.3 (2.1) 3.0 (2.0; 5.0) 0.0; 9.0		0	3.0 (1.9) 2.0 (2.0; 4.0) (1.0; 8.0)	4.1 (2.2) 4.0 (3.0; 5.0) (0; 9.0)	3.4 (0.9) 3.0 (3.0; 4.0) (3.0; 5.0)
* P* value		0.01	Ref	0.06	0.004	0.04
Score Consensus statement						
No ulcers [*n*, (%)]	4 (8.9)		3 (100)	0	1 (5.9)	0
ESGD only [*n*, (%)]	7 (15.6)		0	4 (20.0)	2 (11.8)	1 (20.0)
EGGD only [*n*, (%)]	6 (13.3)		0	4 (20.0)	2 (11.8)	0
ESGD and EGGD [*n*, (%)]	28 (62.2)		0	12 (60.0)	12 (70.6)	4 (80.0)
*P* value		0.02				

*EGUS* Equine Gastric Ulcer Syndrome, *EGGD* Equine Glandular Gastric diseases, *ESGD* Equine Squamous Gastric Disease, *n* number, *oD* other diseases, *TSS* Total Severity Score

**Fig. 4:**
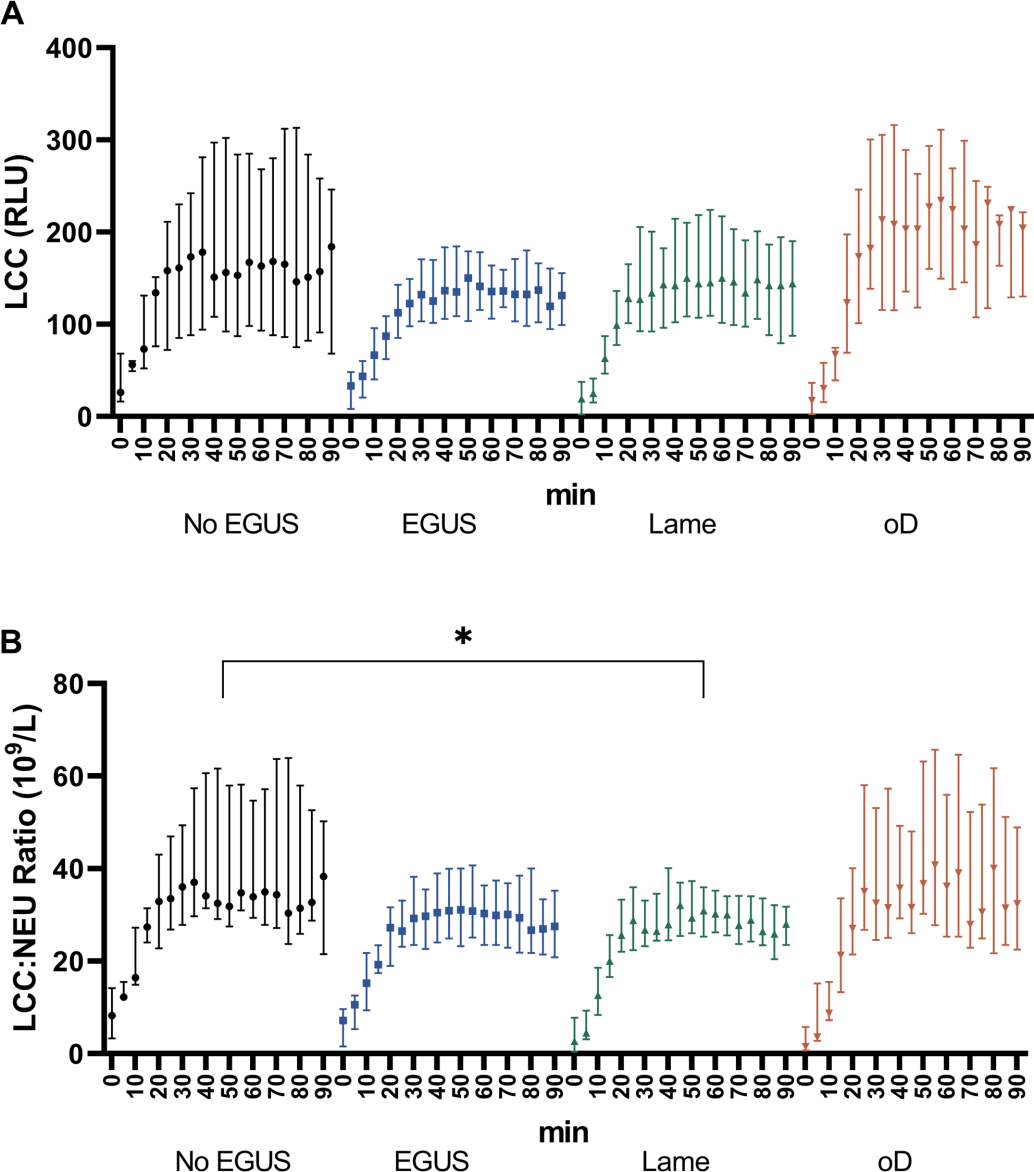
Leukocyte Coping Capacity (LCC, **A**) expressed as relative light units (RLU) and LCC to neutrophilic granulocyte (NEU) count ratio (LCC: NEU ratio, **B**) at initial visit (T1) in the different disease groups. EGUS, Equine Gastric Ulcer Syndrome; oD, other diseases. Symbols represent median and bare represent Q1-Q3 *, *p* < 0.05

The outcome LCC of the model showed that there was no significant difference between the disease groups (Fig. 4A), but that age had to be considered in the models based on the AIC and BIC. Similarly, the AUC_LCC_ did not differ among disease groups, but including age improved the model. The correlation coefficient between the maximal measured LCC value, i.e. LCC peak and AUC_LCC_ was 0.90.

Similarly for the outcome regarding the LCC: NEU ratio (Fig. 4B), the inclusion of age improved the model based on AIC and BIC. The results from our model indicate that LCC: NEU ratio increased with age (*p* = 0.04) and that *Lame* horses had significantly (*p* = 0.012) lower values for LCC: NEU ratio than the horses in the *No EGUS* group. For horses in the *EGUS* or *oD* groups, the model showed no significant difference (*p* > 0.05) in the LCC: NEU ratio compared to *No EGUS* horses. For the LCC: NEU ratio an additional analysis including the TSS in the model, confirmed that *Lame* horses had significant lower LCC: NEU ratio (*p* = 0.03) than the horses in the *No EGUS* group and that LCC: NEU ratio increased with age (*p* = 0.04). Further the AIC and BIC showed that the model including the TSS was slightly better than the model including only age, but the TSS (*p* = 0.50) was not significantly related to the LCC: NEU ratio. For the analysis of AUC_LCC: NEU_, age had to be included in the model but not TSS. When comparing the AUC_LCC: NEU_ there was no statistically significant difference between the disease groups but AUC_LCC: NEU_ increased significantly with age (*p* = 0.04). The correlation coefficient between the maximal measured LCC: NEU ratio and AUC_LCC: NEU_ was 0.92.

### The follow up visit (T2)

Of the 45 horses that were included in the study after the initial gastroscopy, 24 horses were available for follow up gastroscopy 28 days after the initial gastroscopy. The demographic description of this subpopulation is shown in Table 4. Among these horses 10 horses received omeprazole as monotherapy, 13 horses received the combination of omeprazole and sucralfate, including 1 horse for which the owner decided to stop the treatment after 14 days. Finally, one horse received sucralfate as monotherapy for 21 days. The number of horses in each of these treatment groups did not statistically differ among the disease groups at T1 (*p* = 0.51). Further the TSS assessed at T1 was not statistically different in the various treatment groups (*p* = 0.75).

**Table 4:** Demographic description of the population available for a follow up gastroscopy (T2)

			Groups (see Material and Methods)		
	Total		EGUS	Lame	oD
*n*	24		13	8	3
Age (years)					
Mean (SD) median (Q1; Q3) (min; max)	12.7 (6.0) 11.5 (8.5; 18.0) (3.0; 23.0)		10.8 (5.0) 10.0 (8.0; 14.0) (3.0; 21.0)	17.3 (5.9) 20.0 (11.0; 22.0) (9.0; 23.0)	8.7 (4.0) 11.0 (4.0; 11.0) (4.0; 11.0)
*P* value		0.05			
Sex					
g [*n*, (%)]	10 (41.7)		6 (46.2)	2 (25.0)	2 (66.7)
f [*n*, (%)]	14 (58.3)		7 (53.8)	6 (75.0)	1 (33.3)
*P* value		0.53			
BW (Kg)					
Mean (SD) median (Q1; Q3) (min; max)	569.8 (63.7) 577.5 (548.5; 599.5) (377.0; 680.0)		553.8 (78.0) 565.0 (502.0; 590.0) (377.0; 664.0)	576.5 (21.5) 575.0 (562.0; 591.0) (547.0; 609.0)	621.7 (52.0) 605.0 (580.0; 680.0) (580.0; 680.0)
*P* value		0.21			
Breed					
WB [*n*, (%)]	19 (79.2)		8 (61.5)	8 (100.0)	3 (100.0)
TBH [*n*, (%)]	1 (4.2)		1 (7.7)	0 (0.0)	0 (0.0)
STB [*n*, (%)]	1 (4.2)		1 (7.7)	0 (0.0)	0 (0.0)
Haflinger [*n*, (%)]	1 (4.2)		1 (7.7)	0 (0.0)	0 (0.0)
Riding pony [*n*, (%)]	1 (4.2)		1 (7.7)	0 (0.0)	0 (0.0)
Welsh part bred [*n*, (%)]	1 (4.2)		1 (7.7)	0 (0.0)	0 (0.0)
*P* value		1.0			

*BW* Body weight, *EGUS* Equine Gastric Ulcer Syndrome, *f* female, *g* gelding, *n* number, *oD* other diseases, *STB* Standardbred, *THB* Thoroughbred, *WB* Warm blood

At T1 for the 24 horses for whom a follow up visit was available, the *Lame* horses (*n* = 8) had the highest TSS, however the difference was not statistically significant for this subpopulation (Table 5, Appendix A supplementary material Table S3 and Fig. 3 C).

**Table 5:** Severity of the gastroscopic findings at T2 for the 24 horses presented at T2

				Groups as defined in Material and Method		
		Total		EGUS	Lame	oD
	*n*	24		13	8	3
T1	Total severity Score (TSS)					
	Mean (SD) median (Q1, Q3) (min, max)	3,8 (2,1) 3.5 (2,0; 5.0) (1.0; 9.0)		3.6 (2.1) 3,0 (2.0; 5.5) (1.0; 8.0)	4,5 (2.3) 4.0 (3.25; 5.75) (1.0;9.0)	3.0 (0) 3.0 (3.0; 3.0) (3.0; 3.0)
	*P* value (between groups)		0.44	ref	0.53	0.99
	Score Consensus statement					
	No ulcers [*n*, (%)]	0		0	0	0
	ESGD only [*n*, (%)]	0		0	0	0
	EGGD only [*n*, (%)]	4 (16.6)		3 (23.0)	1 (12.5)	0
	ESGD and EGGD [*n*, (%)]	20 (83.3)		10 (77.0)	7 (87.5)	3 (100)
	*P* value (between groups)		1.0			
T2	Total severity Score (TSS)					
	Mean (SD) median (Q1, Q3) (min, max)	2,3 (1,3) 2.0 (1.0; 3.0) (0; 5.0)		2.1 (1.4) 2.0 (1.0; 3.0) (0; 5.0)	2.4 (1.1) 2.5 (1.25; 3.0) (1.0; 4.0)	2,7 (1,5) 3.0 (1.0; 4; 0) (1.0; 4.0)
	*P* value (between groups)		0.75	ref	0.99	0.97
	*P* value (between time points)	0.001		0.05	0.03	0.99
	Score Consensus statement					
	No ulcers [*n*, (%)]	1 (4.2)		1 (7.7)	0	0
	ESGD only [*n*, (%)]	1 (4.2)		1 (7.7)	0	0
	EGGD only [*n*, (%)]	11 (45.8)		7 (53.8)	3 (37.5)	1 (33.3)
	ESGD and EGGD [*n*, (%)]	11 (45.8)		4 (30.8)	5 (62.5)	2 (66.7)
	*P* value (between groups)		0.79			
	*P* value (between time points)		0.08			

*EGUS* Equine Gastric Ulcer Syndrome, *EGGD* Equine Glandular Gastric diseases, *ESGD* Equine Squamous Gastric Disease, *n* number, *oD* other diseases, *TSS* Total Severity Score

At T2 the TSS decreased significantly in all horses (*p* = 0.001, Table 5; Fig. 3B). In the disease group *Lame* this improvement was statistically significant (*p* = 0.03), but not in the other disease groups. Among the 8 horses classified in the *Lame* group at T1 and available for examination at T2, lameness had improved by at least 1 point in 4 cases, while in the other 4 cases, the lameness did not change between T1 and T2. The stomach completely healed in only one horse following treatment, but this horse showed signs of an infectious disease at T2. The proportion of horses showing both ESGD and EGGD decreased between T1 and T2, but the difference did not reach statistical significance. The TSS at T2 did not differ among the treatment groups (p 0.07).

At T2 the owners of 19 horses reported that the horses were still working at the same level as at T1 and that no specific changes in the management had been implemented. The TSS at T2 did not differ significantly in these horses in comparison to the 5 horses (4 horses in the *Lame* group and one horse in the *oD* group) that had been rested (*p* = 0.09).

In the bivariate mixed effects model for T2 LCC: NEU ratio was significantly lower in the *Lame* group (*p* = 0.031) in comparison to the *EGUS* and *oD* groups independent of the additional predictors. The model which included treatment was the best model based on AIC und BIC.

## Discussion

Concerning the LCC measurements, the main finding of our study is that the LCC: NEU ratio at T1was significantly lower in the *Lame* group at T1. Also, *Lame* horses or those with other diseases (*oD* group) had the highest prevalence of both ESGD and EGGD and had the highest TSS. Although our study setting cannot determine whether the lameness or the presence of EGUS was responsible for the lower LCC, the results of horses in the *Lame* group support our hypothesis and indicate a possible link between lameness, EGUS, and stress. Indeed, in horses with a condition leading to a higher level of stress and discomfort, the LCC: NEU ratio decreased in relation to the predicted stress level. The pain caused by the orthopedic problem and associated stress may also have led to more severe gastric ulcers. However, further studies should include horses which present with a primary orthopedic problem and no evidence of occurrence of EGUS. The LCC: NEU ratios decreased also in the disease groups *EGUS* and *oD*, however, these differences in LCC: NEU ratios were not statistically significant. Horses in these groups were similarly expected to experience a certain level of stress as these horses showed gastric ulcers that, especially in the group *oD*, were often a combination of ESGD and EGGD. However, as this difference in LCC: NEU ratios was not statistically significant, we could not confirm our hypothesis for these disease groups. The lack of statistical significance may, however, be partly attributable to the relatively low sample size in the different disease groups and the rather large interquartile ranges in the *oD* group in particular. However, despite not being statistically significant this difference may very well represent a biologically meaningful difference in stress load and the horse’s ability to cope with stress. Orthopedic diseases have been shown to be associated with pain, gait abnormalities and behavioral changes during exercise [12–14, 46, 47]. Moreover, changes in behavior could even be shown in anticipation of the pain expected to be experienced during exercise [59]. Chronic pain can alter nociceptive pathways and lead to a state of sensitization and up-winding of pain sensation [60]. If not recognized [48–50] and subsequently not presented for veterinary care, orthopedic pain might become chronic. Due to the pain sensitization, chronic orthopedic diseases might lead to higher stress load compared to more short-term disease states or discomfort, as was assumed to have been present in the groups *oD* or *EGUS*. By introducing the concepts of allostasis (i.e., the physiological and behavioral adjustments in response to stressors to “achieve stability through change”) and allostatic load (i.e., the cumulative cost incurred by somatic systems due to repeated or chronically activated allostasis), it can be explained how differences of intensity and duration of stress affects the stress response as well as the associated biological costs [15–18, 61]. Therefore, a higher allostatic load in horses with lameness possibly manifested in the reduced LCC: NEU ratio measured in our study population. Our results support the assumption that orthopedic diseases, in particular, might represent a substantial and sustained stress load and are a serious health and welfare concern [62]. These findings, however, must be confirmed by future studies including additional stress indices and biomarkers such as cortisol levels.

Another finding of or study was the association between age and the function of the NEU. The LCC: NEU increased with age and the statistical models improved when age was included. It is widely acknowledged that the impact of stress on the equine body is influenced by various factors, including the nature of the stressor—its intensity, duration, frequency, predictability, controllability, and avoidability—as well as the horse’s breed, age, and prior exposure to stressful stimuli or anticipation thereof [63–68]. Notably, younger horses exhibit heightened stress reactivity compared to adult horses [69]. However, age had no negative effect on LCC in our study population. The LCC increased with age and the enhanced LCC even remained significant once LCC was corrected for the number of neutrophils (i.e., LCC: NEU ratio). We can, therefore, exclude a potential mass effect attributable to a shift in blood cell subpopulations with age. Other studies have shown that age affects both the innate and the adaptive immune systems, the ensuing reduced function of which is called immunosenescence [70, 71]. Even though the topic as not been as profoundly studied as in humans, available literature suggests that in horses the total lymphocyte count, along with their subpopulations, decreases with age [70–74]. It is also reported that lymphocytes show a lower proliferation capacity and that the antibody production and therefore the response to vaccination seems to be affected by immunosenescence [70, 71, 73, 75]. However, cytokine production by peripheral blood mononuclear cells (PBMC) of older horses appears to increase as shown with a greater proportion of INF-g and TNF-a positive cells [76, 77] and higher INF-g, Il-1b, Il-6 and Il-8 expression by PBMC in aged horses than in younger horses [78, 79]. This activation of the immune system leads to a pro-inflammatory stage described as inflammaging. The innate immune system seems to be less affected by age in horses [70]. It has been shown that the oxidative burst capacity assessed in flowcytometry seems not to be affected by increasing age when comparing horses with an average age of 10 years to horses with an average age of 23 years and chemotactic ability was even increased in the group of older horses [80]. However, another study comparing slightly different age groups (mean age 13 years vs. 22 years) found a reduced concentration of myeloperoxidase, a marker of neutrophil degranulation in the plasma of older horses [81]. The moderate increase in LCC and slight increase in LCC: NEU ratio with age in our study indicate that the ability of NEU to react to the stimulation with PMA in the point of care-methods used was intact as shown in the study of McFarlane et al. [80]. However, comparisons with former studies are difficult because some of the discrepancies could be explained by the use of different methods. Further, the mean age of our study population was 11.6 ± 6.0 years and therefore corresponds more to the younger group in the former studies [80, 81]. Yet some of the horses were older (oldest horse was 23 years old, especially in the *Lame* group) and they might have an altered function of NEU. Therefore, age should be considered when assessing LCC in horses. An increase in LCC: NEU ratio could also be related to a reduced number of NEU but the NEU in our population was within reference range and NEU number seemed not to be affected by age [72, 73, 81].

In addition to age, BCS might have an influence on the function of the immune system. Obese horses showed higher oxidative burst capacity independently of whether they were insulin dysregulated or not [82, 83], and further oxidative burst capacity positively correlated with insulin concentration [83]. However, the effect of obesity on the function of the immune system could not be clearly differentiated from the effect of age [77, 83]. In our study, the BCS did not differ among disease groups and was quite homogeneous with only 1 horse with a BCS of >7 (Appendix A supplementary material Table S1), in contrast to the differences in age where the *Lame* horses had a tendency to be older. However, BCS and metabolic status should be assessed in further studies on immune function.

These considerations are in line with the known multidimensional interaction between the immune system, the endocrine system and the nervous system [15, 16, 84, 85]. This complex interaction explains the effect of an activated HPA axis on the immune system, as well as how an activated immune system can activate a stress response. The effect can be regulated by direct interaction with receptors on the immune cells [16, 84, 85] or by a modification of inflammatory cytokine production (INF-g, Il-1,Il-1B, Il-2, Il-6, TNF) [15, 16]. These complex interfaces must be understood in the context of the ability of an organism to adapt to stressors in its surrounding environment. Every challenge to its homeostasis will produce a reaction with the goal to maintain or reestablish homeostasis. These dynamic physiological changes (i.e. allostasis) and the number and intensity of these dynamic changes (i.e. allostatic load) are directly linked to the pathophysiological costs of coping with stressors [15, 18, 61]. Concerning the immune system this concept is illustrated by the fact that in cases of short-term stress the immune system might be enhanced in order to improve the protection against invading microorganism and thereby contribute to better immunoprotection. During long-term stress, however, the immunopathologic or immunosuppressive effect predominates [17]. Short-term exposure to psychological stress also showed enhanced NEU activation in humans [86]. In an equine study a pro-inflammatory state with increased serum concentration of cytokines TNF-a and Il-6 has been reported in horses with EGUS alone or following administration of non-steroidal anti-inflammatory drugs (NSAID) due to concomitant diseases, in comparison to healthy horses [87]. However, in this study it was not reported how long the concomitant diseases lasted and how many horses in the *EGUS* group had been treated for these primary diseases [87]. It may be that these primary diseases represent the primary stress challenge for the organism [10] and could therefore have led to the activation of the immune system and secondarily to EGUS.

With prolonged duration of a stressful stimulus, a negative feedback loop in the HPA will assure that cortisol exercises a suppressive effect on the immune response [17]. However, the preexisting allostatic load (leading to the current physiological state and ability to cope with stress) that the organism has already faced also has an impact on the effect of stress hormones in the stress response [15, 61], and immuno-dysregulation towards a prepathological state becomes likely in such context [17]. It is commonly accepted that a state of stress is involved in the manifestation of ESGD and EGGD [36, 43]. Likewise, a state of long-lasting stress is present in horses with chronic orthopedic diseases and associated pain [13, 47]. Even if the timeline for stress load could not be fully assessed in our field observational clinical study our results based on LCC support these findings in the literature - with a significantly lower value for LCC, which, in the case of normalizing stress levels would show a higher level for LCC. This decrease in LCC was particularly clear in horses presented with lameness, a state known to be associated with pain [13, 47].

The majority of studies in the scientific literature have reported on the potential use of LCC to assess short-term or acute stress in the context of animal capture and transport scenarios [19, 20, 24]. Gormally & Romero (2020) [88] reported LCC to reflect short-term or acute stress and suggested that additional studies should assess LCC as a marker for longer, sustained stress. NEUs are not only equipped with receptors sensitive to hormones involved in the stress response but also possess receptors which are sensitive to disease-associated stress signals including changes in blood biochemistry, red cell hemodynamics and in cytokine levels [16, 84, 85, 89]. This sensitivity to several stress mediators and disease-related physiological changes make NEUs excellent indicators in evaluating the pathophysiological (internal) state of the animal and the resulting ability to cope with the (external) environment. We are, therefore, convinced that LCC holds particularly promising potential not only for assessing stress caused by external factors, as shown in the context of wildlife manipulations or housing [25–28, 90], but also for evaluating stress load in the context of diseases [21, 35]. However, more studies in this direction are warranted to better understand LCC patterns and to establish thresholds for different equine stress related diseases.

Regarding the methodology used, it is important to point out that the LCC measurement focuses on the innate immune system in general, and on the function of NEU in particular. As part of the immune system a stress response modifies this reaction as NEU function is directly influenced by cortisol [16] or catecholamines [84] as well as other mediators. When NEU are activated by contact with pathogens, they start their phagocytic activity and increase the production and release of ROS during the oxidative burst [21, 22]. The addition of synthetic substances as PMA facilitates and enhances the production of ROS [22, 23]. In contrast to other methods [80, 81] the portable chemiluminometer enabled the measurement of the LCC in one step within 30 min of blood collection. Therefore, the blood did not need to be shipped or stored nor processed after collection, which is time consuming and could alter the NEU function. The method used in this study can be easily performed in the clinical setting as well as stall-side and is easier and faster than the other formerly described methods with a long incubation period and/or time consuming isolation methods for subsequent PCR reaction or flowcytometry [80, 81]. Further, a time profile of the activation of the NEU can be established over the 90 min of measurement, this shows the dynamism of the reaction of the NEU to the stimulation. Such time profiles allow calculation of an AUC as used in other studies [24–26, 33]. The AUC in our analysis was highly correlated to maximal measured LCC and LCC: NEU ratio, as previously shown for mammals such as deer [24] or rhinoceros [91]. Analyses of AUC confirmed the influence of age on the LCC and LCC: NEU ratio, however did not demonstrate a difference in disease groups in our study sample in contrast to a study in bears, were AUC and LCC were interchangeable [25]. A slightly disadvantageous aspect of the method is that, as the chemiluminometer measures the total fluorescence produced in the sample the number of NEU in the sample needs to be known in order to correct RLU for the mass effect and to have a more accurate estimation of oxidative burst capacity of the NEU [20, 24, 26].

The peak activation of the NEU with the concentration of PMA used seemed to occur later in horses compared to other big mammals. Indeed in pigs [34], badgers [20], bears [25] or deer the maximum of LCC was measured between 10 and 15 min after starting the reaction, which is much shorter than the 50–60 min in horses seen in our study.

Even if the concentration of luminol was similar in all studies the concentration of PMA varied between 10^− 4^ mol/L and 10^− 6^ mol/L in most of the studies [20, 25–28, 33, 34]. Especially in non-human primates a marked difference of reactivity could be seen by increasing the concentration of PMA to 10^− 3^ mol/L [26]. Therefore, further studies should test whether better discrimination between stress level or disease groups in horses would be achieved by using higher concentrations of PMA as seen in non-human primates [26]. Similarly, the temperature for the reaction should be optimized. Former studies used 37.0 °C for the reaction [20, 25–28, 32–34], whereas we used 37.5 °C. This temperature is at the lower range of physiological IBT of horses [92, 93]. A better reaction and discrimination between stress level or disease groups might be obtained with slightly higher temperature that is more representative of the average physiological IBT of horses.

A further finding of our study was that the healing rate for the gastric ulcers was poor in our study population, even if the TSS decreased in all horses and significantly in the *Lame* horses. Only one horses had complete healing of the gastric ulcers after the 28 days of treatment but showed clinical signs of respiratory tract disease at follow-up examination (nasal discharge, cough and a slight increase in abdominal respiratory effort). Some of the horses were still at work at their previous level at T2. They showed similar or only slight improvement in orthopedic problems or had other diseases. Therefore, one of the reasons for the poor response was certainly the difficulty in addressing concomitant diseases and to implement management changes for the study population due to lack of compliance to advice in the management of EGUS, for example [94–96]. To account for this fact, we tried to subjectively assess the change in stress level between the two visits by classifying the stress level subjectively, however, the inclusion of stress level did not improve the model.

Finally, we used a new way to grade the EGUS in addition to the grading system proposed by the consensus statement and adapted for the EGGD [36, 54]. Another numeric grading system for EGGD [55] has recently been proposed to better assess the lack of healing in EGGD. We combined this recently published score with the ESGD score in order to facilitate the comparison before and after treatment. The TSS seemed to be easy to use and provided confirmation of the higher presence of EGGD and ESGD in lame horses. Further studies are needed to validate this new score.

The study had several limitations. Our study was a clinical study with horses that were primarily presented for gastroscopy. In the given context of private practice, it was not possible and/or justified to perform gastroscopy or to take additional blood samples on healthy horses. Further, due to logistical and financial constraints, the presence of metabolic diseases such as pituitary pars intermedia dysfunction (PPID) or equine metabolic syndrome (EMS) [70] was only excluded by clinical examination. However, with a clinically founded suspicion, further examinations were proposed to the owners. Therefore, influence of an underlying condition not yet clinically apparent could have influenced the reaction of the NEU [70]. In particular, an immunosuppressive effect of PPID has been shown, although the results concerning the influence of PPID on NEU function were conflicting [70, 79, 80, 97]. With EMS, the association between the disease and the immune system is more complex [70, 98]. In one study, hyperinsulinemic obese horses showed an enhanced oxidative burst activity of NEU in vitro and increased insulin concentration was positively correlated with neutrophilic oxidative burst [83]. In other studies, it was more difficult to differentiate between the effect of age, obesity or concurrent PPID [77, 83, 99]. Therefore, such metabolic diseases should be excluded in further studies by appropriate laboratory tests. Another limitation is that the horses were assessed for lameness by trotting in hand. This method may not be sensitive enough to detect more subtle forms of lameness (e.g., bilateral or multi-limb lameness, neck pain, or back pain) which could be sources of pain-induced stress. Therefore, we cannot exclude the possibility that horses with these types of orthopedic diseases may have been misclassified into other groups. However, the horses were primarily presented for gastroscopy, and the lameness revealed had mostly gone unrecognized by the owners, highlighting the reported discrepancy between owner and veterinarian assessments of gait abnormalities [48–50]. In any case, measuring LCC in horses with a primary complaint of orthopedic disease would be an interesting and important future step in assessing LCC as a biomarker of stress. Similarly, measuring cortisol concentrations as the most established stress marker and correlating it to LCC would be interesting for future studies. Likewise, to compare and correlate LCC to any other marker within the HPA-axis or sympathetic nervous system or behavioral scores would allow better understanding of the stress response in horses.

Further, the small sample size, especially in some of the diseases groups (i.e. group *No EGUS* or *oD*) and in the group of horses where a follow-up exam was available, may limit the power of our results and the outcomes should be taken with caution and cannot be generalized. Another limitation of the presented study is that the classification of the disease groups could not be based on more detailed investigations due to the fact that further diagnostic investigations for the primary identified clinical problem had to be differed to a different time due to the time constraints of an ambulatory practice or were declined by the owners. Better compliance of the owners to the implementation of veterinary advice would have certainly contributed to more meaningful results for T2. Also, the population of horses was not homogeneous with a large variety of ages and breeds. We recommend focusing on a more homogeneous study population in future studies. Similar, the low compliance for advised management changes was disappointing and certainly affected the healing rate of the ulcers. Only one horse showed complete healing. It is well known that especially ESGD can be favored by stress associated with several management factors (e.g. high grain and starch diets, low turnout to pasture, no permanent access to water, long transport, little social contact [38, 39]) and high-intensity training [40–42]. Also, recent studies have suggested that horses with EGGD may have a higher stress sensitivity and react with higher cortisol levels both in plasma after a novel object test and in saliva after an ACTH stimulation test [44, 45]. Therefore, stress or allostatic load seems to be an important pathophysiological factor in both forms of EGUS [36, 43] and our results highlight the importance of adapting the housing and management conditions of horses towards stress reduction in the successful management of this condition.

## Conclusion

In conclusion, horses in our study population that exhibited both lameness and gastric ulcers at T1 had a low LCC: NEU ratio. Despite the limitations of the study, these findings indicate a possible link between lameness, EGUS, and stress. The results of this preliminary study support further studies assessing LCC as an indicator for stress or allostatic load, however other potential stress parameters should also be included. Additionally, LCC measurements could be expanded to include other common equine diseases.

## Supplementary Information

Supplementary Material 1: Appendix B supplementary material Figure S1-S6

Supplementary Material 2: Appendix A supplementary material Table S1-S4

## Data Availability

The datasets used and/or analyzed during the current study are available from the corresponding author on reasonable request.

## Abbreviations

ACTH: Adrenocorticotropic Hormone
AIC: Akaike’s Information Criterion
ALB: Albumin
ALKP: Alkaline Phosphatase
AST: Aspartate-aminotransferase
AUC: Area Under the Curve
BCS: Body Condition Score
BIC: Bayesian Information Criterion
BUN: Blood Urea Nitrogen
BW: Bodyweight
Ca: Calcium
CK: Creatine Kinase
CREA: Creatinine
DMSO: Dimethyl Sulfoxide
ECEIM: European College of Equine Internal medicine
EGGD: Equine Glandular Gastric Disease
EGUS: Equine Gastric Ulcer Syndrome
EMS: Equine Metabolic Syndrome
ESGD: Equine Squamous Gastric Disease
f: Female
g: Gelding
GGT: Gamma-glutamyl transferase
GLOB: Globulins
GLU: Glucose
HPA: Hypothalamic-Pituitary-Adrenal axis
HR: Heart Rate
HRV: Heart Rate Variability
IBT: Inner Body Temperature
LC: Leukocyte
LCC: Leukocyte Coping Capacity
LCC: NEU: ratio LCC divided by the NEU count
LDH: Lactatdehydrogenase
n: Number
NEU: Neutrophil granulocytes
NSAID: non-steroidal anti-inflammatory drugs
oD: Other diseases
PBS: Phosphate-buffered saline
RLU: Relative Light Units
PBMC: Peripheral Blood Mononuclear Cells
PMA: Phorbol Myristate Acetate
PPID: Pituitary Pars Intermedia Dysfunction
Q1: First quantile
Q3: Third quantile
ROS: Reactive Oxygen Species
RR: Respiratory Rate
SD: Standard Deviation
STB: Standardbred
TBIL: Total Bilirubin
THB: Thoroughbred
TP: Total Protein
TSS: Total Severity Score
WB: Warm Blood
