# Supplementary material for: Preliminary assessment of the leukocyte coping capacity as a point of care marker in horses with stress associated diseases
Source: BMC Vet Res. 2025 Dec 7;22:199. doi: 10.1186/s12917-025-05179-9 (PMC13049875; doi:10.1186/s12917-025-05179-9)
Supplement: Supplementary file 1 — Supplementary Material 1: Appendix B supplementary material Figure S1-S6 [file 12917_2025_5179_MOESM1_ESM.docx]

**Appendix B Supplementary Material**

**Figure S1**: Hematological parameters of the study population at T1 (*n*=45). EGUS, Equine Gastric Ulcer Syndrome; HGB, Hemoglobin; HTC, Hematocrit; oD, Other diseases; PLT, Platelets; RBC, Red Blood Cells. The dashed lines represent the reference values as defined by the laboratory method used.

**Figure S2**: Differential cell count of the study population at T1 (*n*=45). EGUS, Equine Gastric Ulcer Syndrome; EOS, Eosinophilic granulocytes; LYM, Lymphocytes; MONO, Monocytes; oD, Other diseases. The dashed lines represent the reference values as defined by the laboratory method used.

**Figure S3**: Results of the biochemistry analyses of the study population at T1 (*n*=45). ALB, Albumin; EGUS, Equine Gastric Ulcer Syndrome; GLOB, Globulin; oD, Other diseases; TP, Total protein. The dashed lines represent the reference values as defined by the laboratory method used.

**Figure S4:** Results (continued) of the biochemistry analyses of the study population at T1 (*n*=45). ALKP, Alkaline phosphatase; AST, Aspartate-aminotransferase; EGUS, Equine Gastric Ulcer Syndrome; GGT, Gamma-glutamyl-transferase; oD, Other diseases; TBIL, Total bilirubin. The dashed lines represent the reference values as defined by the laboratory method used.

**Figure S5:** Results (continued) of the biochemistry analyses of the study population at T1 (*n*=45). BUN, Blood urea nitrogen; CK, Creatine kinase; CREA, Creatinine; EGUS, Equine Gastric Ulcer Syndrome; LDH, Lactate dehydrogenase; oD, Other diseases. The dashed lines represent the reference values as defined by the laboratory method used.

**Figure S6:** Results (continued) from the biochemistry analyses of the study population at T1 (*n*=45). Ca, Calcium; EGUS, Equine Gastric Ulcer Syndrome; GLU, Glucose; oD, Other diseases. The dashed lines represent the reference values as defined by the laboratory method used.
