# Supplementary material for: Preliminary assessment of the leukocyte coping capacity as a point of care marker in horses with stress associated diseases
Source: BMC Vet Res. 2025 Dec 7;22:199. doi: 10.1186/s12917-025-05179-9 (PMC13049875; doi:10.1186/s12917-025-05179-9)
Supplement: Supplementary file 2 — Supplementary Material 2: Appendix A supplementary material Table S1-S4 [file 12917_2025_5179_MOESM2_ESM.docx]

**Appendix A Supplementary material**

**Table S1:** Physiological parameter of the study population at T1

|  | | **Total** |  | **Groups (see Material and Methods)** | | | |
| --- | --- | --- | --- | --- | --- | --- | --- |
|  | |  |  | **No EGUS** | **EGUS** | **Lame** | **oD** |
| *n* | | 45 |  | 3 | 20 | 17 | 5 |
| **BCS** | |  |  |  |  |  |  |
| Mean (SD)  Median (Q1; Q3)  min; max | | 5.87 (0.79)  6.0 (5.0; 6.0)  4.0; 8.0 |  | 6.33 (0.57)  6.0 (6.0; 7.0)  6.0; 7.0 | 6.15 (0.59)  6.0 (6.0; 6.0)  5.0; 8.0 | 5.53 (0.94)  5.0 (5.0; 6.0)  4.0; 7.0 | 5.60 (0.55)  6.0 (5.0; 6.0)  5.0; 6.0 |
|  | ***P* value** |  | 0.05 | ref | 0.99 | 0.25 | 0.43 |
| **IBT** (°C) | |  |  |  |  |  |  |
| Mean (SD)  Median (Q1; Q3)  min; max | | 37.1 (0.3)  37.1 (37.0;37.4)  36.3; 37.8 |  | 37.2 (0.4)  37.1 (36.8; 37.6)  36.8; 37.6 | 37.1 (0.3)  37.1 (36.9; 37.3)  36.3; 37.6 | 37.3 (0.3)  37.2 (37.0; 37.4)  36.8; 37.8 | 37.1 (0.1)  37.2 (37.0; 37.2)  37.0; 37.2 |
|  | ***P* value** |  | 0.53 | ref | 0.99 | 0.99 | 0.99 |
| **HR** (bpm) | |  |  |  |  |  |  |
| Mean (SD)  median (Q1; Q3)  min; max | | 45 (10)  43 (37; 48)  32; 88 |  | 49 (10)  48 (40; 60)  40; 60 | 47 (13)  42 (40; 48)  36; 88 | 42 (6)  42 (36; 48)  32; 50 | 43 (6)  44 (37; 48)  33; 48 |
|  | ***P* value** |  | 0.54 | ref | 0.99 | 0.60 | 0.99 |
| **RR** (rpm) | |  |  |  |  |  |  |
| Mean (SD)  Median (Q1; Q3)  min; max | | 14 (3)  13 (11; 16)  8; 20 |  | 11 (1)  12 (10; 12)  10; 12 | 13 (3)  13 (10; 16)  8; 20 | 14 (3)  14 (12; 18)  10; 20 | 15 (2)  16 (13; 17)  12; 18 |
|  | ***P* value** |  | 0.24 |  | 0.99 | 0.43 | 0.23 |

BCS, Body Condition Score; BW, Body weight; IBT, Inner Body Temperature; HR, Heart Rate; *n*, number; oD, other diseases; RR, Respiratory Rate.

**Table S2:** Clinical signs, laboratory findings and diagnoses of horses in the group other diseases (oD)

| **Case number** | **Clinical signs** | **Laboratory findings** | | | **Diagnosis** |
| --- | --- | --- | --- | --- | --- |
|  |  |  | | **Reference range** |  |
|  |  |  | | LC: 5.5-12.5 10^9^/L  NC: 3.0-7.0 10^9^/L |  |
| **1** | Nasal discharge  Swollen submandibular lymph nodes | Normal LC count | | | Acute infection  of the respiratory tract |
|  |  |  | LC: 8.4 10^9^/L  NEU: 5.2 10^9^/L | |  |
|  |  |  | | |  |
| **2** | Increased respiratory rate | Increased LC count | | | Acute infection  of the respiratory tract |
|  |  |  | LC: 12.4 10^9^/L  NEU: 8.4 10^9^/L | |  |
|  |  |  | | |  |
| **3** | Increased respiratory rate, slight nasal discharge | Increased LC count | | | Acute infection  of the respiratory tract |
|  |  |  | LC: 14.7 10^9^/L  NEU: 11.1 10^9^/L | |  |
|  |  |  | | |  |
| **4** | Loos faeces  positive faecal sample for intestinal parasites | Low LC count | | | Parasitism |
|  |  |  | LC: 5.3 10^9^/L  NEU: 3.5 10^9^/L | |  |
|  |  |  | | |  |
| **5** | Loos faeces  positive faecal sample for intestinal parasites | Low LC count | | | Parasitism |
|  |  |  | LC: 3.5 10^9^/L  NEU: 2.1 10^9^/L | |  |

LC, Leucocyte, NEU, Neutrophilic granulocyte

**Table S3:** Detailed diagnosis at Gastroscopy at T1

|  | | **Groups (see Material and Methods)** | | | | | | | | | | | | | | | |  |  |
| --- | --- | --- | --- | --- | --- | --- | --- | --- | --- | --- | --- | --- | --- | --- | --- | --- | --- | --- | --- |
|  | | **No EGUS** | | | **EGUS** | | | | **Lame** | | | | **oD** | | | | |  |  |
|  | | ***n*** | Total New Score | Total Severity Score | **EGGD** | ***n*** | Total New Score | Total Severity Score | **EGGD** | ***n*** | Total New Score | Total Severity Score | **EGGD** | ***n*** | | Total New Score | Total Severity Score | **Total *n*** |  |
|  | | **3** |  | |  | **20** |  |  |  | **17** |  |  |  | | **5** |  |  | **45** |  |
| No ulcers [*n*, (%)] | | 3  (100) |  | |  | 0 |  |  |  | 1 (5.9) |  |  |  | |  |  |  | 4  (8.9) |  |
| ESGD only [*n*, (%)] | | 0 |  | |  | 4 (20) |  |  |  | 2 (11.8) |  |  |  | | 1 (20) |  |  | 7 (15.6) |  |
| EGGD only [*n*, (%)] | | 0 |  | |  | 4  (20) |  |  |  | 2  (11.8) |  |  |  | | 0 |  |  | 6 (13.3) |  |
| ESGD and EGGD  [*n*, (%)] | | 0 |  | |  | 12  (60) |  |  |  | 12  (70.6) |  |  |  | | 4 (80) |  |  | 28 (62.2) |  |
| ***P* value** | |  |  | |  |  |  |  |  |  |  |  |  | |  |  |  |  | 0.02 |
| Mean (SD)  Median (Q1; Q3)  min; max | |  |  | 0 |  |  |  | 3.0 (1.9)  2.0 (2.0; 4.0)  1.0; 8.0 |  |  |  | 4.1 (2.2)  4.0 (3.0; 5.0)  0; 9.0 |  |  | |  | 3.4 (0.9)  3.0 (3.0; 4.0)  3.0; 5.0 |  |  |
| ***P* value** | |  | ref | |  |  |  | 0.06 |  |  |  | 0.004 |  | |  |  | 0.04 |  | 0.01 |
| **ESGD** | **SS-0** | **3** |  | 0 |  |  |  |  |  | **1** |  | 0 |  |  | |  |  | **4** |  |
|  |  |  |  | 0 |  |  |  |  |  |  |  |  |  |  |  |  |  |  |  |
|  |  |  |  | 0 |  |  |  |  |  |  |  |  |  |  |  |  |  |  |  |
|  |  |  | | | **GS-1** | **1** | 2 | 2 | **GS-1** |  |  |  | **GS-1** | |  |  |  | **1** |  |
|  |  |  |  |  | **GS-2** | **3** | 1 | 1 | **GS-2** | **2** | 1 | 1 | **GS-2** | |  |  |  | **5** |  |
|  |  |  |  |  |  |  | 2 | 2 |  |  | 2 | 2 |  |  |  |  |  |  |  |
|  |  |  |  |  |  |  | 2 | 2 |  |  |  |  |  |  |  |  |  |  |  |
|  | **SS-1** |  | | |  | **3** |  | 1 |  |  |  |  |  | |  |  |  | **3** |  |
|  |  |  |  |  |  |  |  | 1 |  |  |  |  |  |  |  |  |  |  |  |
|  |  |  |  |  |  |  |  | 1 |  |  |  |  |  |  |  |  |  |  |  |
|  |  |  | | | **GS-1** | **2** | 1 | 2 | **GS-1** | **1** | 2 | 3 | **GS-1** | |  |  |  | **3** |  |
|  |  |  |  |  |  |  | 1 | 2 |  |  |  |  |  |  |  |  |  |  |  |
|  |  |  |  |  | **GS-2** | **1** | 1 | 2 | **GS-2** | **1** | 2 | 3 | **GS-2** | | **3** | 2 | 3 | **5** |  |
|  |  |  |  |  |  |  |  |  |  |  |  |  |  |  |  | 2 | 3 |  |  |
|  |  |  |  |  |  |  |  |  |  |  |  |  |  |  |  | 2 | 3 |  |  |
|  |  |  |  |  | **GS-3** |  |  |  | **GS-3** | **1** | 6 | 7 | **GS-3** | |  |  |  | **1** |  |
|  | **SS-2** |  | | |  | **1** |  | 2 |  | **1** |  | 2 |  | |  |  |  |  | **2** |
|  |  |  | | | **GS-1** | **3** | 1 | 3 | **GS-1** | **1** | 2 | 4 | **GS-1** | |  |  |  | **4** |  |
|  |  |  |  |  |  |  | 2 | 5 |  |  |  |  |  |  |  |  |  |  |  |
|  |  |  |  |  |  |  | 6 | 8 |  |  |  |  |  |  |  |  |  |  |  |
|  |  |  |  |  | **GS-2** | **4** | 1 | 3 | **GS-2** | **2** | 3 | 5 | **GS-2** | |  |  |  | **6** |  |
|  |  |  |  |  |  |  | 1 | 3 |  |  | 4 | 6 |  |  |  |  |  |  |  |
|  |  |  |  |  |  |  | 2 | 4 |  |  |  |  |  |  |  |  |  |  |  |
|  |  |  |  |  |  |  | 4 | 6 |  |  |  |  |  |  |  |  |  |  |  |
|  |  |  |  |  | **GS-3** | **1** | 4 | 6 | **GS-3** |  |  |  | **GS-3** | |  |  |  | **1** |  |
|  | **SS-3** |  | | |  |  |  |  |  | **1** |  |  |  | | **1** |  | 3 | **2** |  |
|  |  |  | | | **GS-1** |  |  |  | **GS-1** | **3** | 1 | 4 | **GS-1** | | **1** | 2 | 5 | **4** |  |
|  |  |  |  |  |  |  |  |  |  |  | 1 | 4 |  |  |  |  |  |  |  |
|  |  |  |  |  |  |  |  |  |  |  | 2 | 5 |  |  |  |  |  |  |  |
|  |  |  |  |  | **GS-2** |  |  |  | **GS-2** | **2** | 2 | 5 | **GS-2** | |  |  |  | **2** |  |
|  |  |  |  |  |  |  |  |  |  |  | 6 | 9 |  |  |  |  |  |  |  |
|  |  |  |  |  | **GS-3** |  |  |  | **GS-3** |  |  |  | **GS-3** | |  |  |  |  |  |
|  | **SS-4** |  | | |  |  |  |  |  |  |  |  |  | |  |  |  |  |  |
|  |  |  | | | **GS-2** | **1** | 1 | 5 | **GS-2** | **1** | 2 | 6 | **GS-2** | |  |  |  | **2** |  |

EGUS, Equine Gastric Ulcer Syndrome; EGGD, Equine Glandular Gastric Disease; ESGD, Equine Squamous Gastric Disease; *n*, number; oD, other diseases; GS, Glandular Score; SS, Squamous Score; TSS, Total Severity Score

**Table S4:** Detailed diagnosis at Gastroscopy at T2

|  | | **Groups (see Material and Methods)** | | | | | | | | | | | | | | | | | | | |  | |
| --- | --- | --- | --- | --- | --- | --- | --- | --- | --- | --- | --- | --- | --- | --- | --- | --- | --- | --- | --- | --- | --- | --- | --- |
|  | | **EGUS** | | | | | | **Lame** | | | | | **oD** | | | | | | | | |  | |
|  | |  | ***n*** | Total New Score | Total Severity Score | | Gastroscopic assessment |  | ***n*** | Total New Score | Total Severity Score | Gastroscopic assessment |  | ***n*** | | Total New Score | | Total Severity Score | | | Gastroscopic assessment | **Total *n*** |  |
|  | | **EGGD** | **13** |  |  |  | | **EGGD** | **8** |  |  |  | **EGGD** | | **3** | |  | |  |  | | **24** | |
| **ESGD** | **SS-0** |  | **1** | 0 | 0 | **I (oD)** | |  |  |  |  |  |  | |  | |  | |  |  | | **1** | |
|  |  | **GS-1** | **2** | 1 | 1 | **I** | | **GS-1** | **1** | 1 | 1 | **No** | **GS-1** | | **1** | | 1 | | 1 | **I** | | **5** | |
|  |  |  | **1** | 2 | 2 |  |  |  |  |  |  |  |  |  |  |  |  |  |  |  |  |  |  |
|  |  | **GS-2** | **2** | 1 | 1 | **I** | | **GS-2** | **2** | 1 | 1 | **I** | **GS-2** | |  | |  | |  |  | | **6** | |
|  |  |  | **1** | 2 | 2 | **I (L)** | |  |  | 3 | 3 | **No** |  |  |  |  |  |  |  |  |  |  |  |
|  |  |  | **1** | 3 | 3 | **W** | |  |  |  |  |  |  |  |  |  |  |  |  |  |  |  |  |
|  | **SS-1** |  | **1** | 1 | 2 | **W** | |  |  |  |  |  |  | |  | |  | |  |  | | **1** | |
|  |  | **GS-1** |  |  |  |  | | **GS-1** | **1** | 1 | 2 | **I** | **GS-1** | | **1** | | 2 | | 3 | **No** | | **2** | |
|  |  | **GS-2** | **3** | 2 | 3 | **W** | | **GS-2** | **1** | 2 | 3 | **I** | **GS-2** | |  | |  | |  |  | | **4** | |
|  |  |  |  | 2 | 3 | **I** | |  |  |  |  |  |  |  |  |  |  |  |  |  |  |  |  |
|  |  |  |  | 3 | 4 | **I** | |  |  |  |  |  |  |  |  |  |  |  |  |  |  |  |  |
|  |  | **GS-3** | **1** | 4 | 5 | **I (L)** | | **GS-3** | **1** | 3 | 4 | **I** | **GS-3** | |  | |  | |  |  | | **2** | |
|  | **SS-2** |  |  |  |  |  | |  |  |  |  |  |  | |  | |  | |  |  | |  | |
|  |  | **GS-1** |  |  |  |  | | **GS-1** | **1** | 0 | 2 | **I** | **GS-1** | |  | |  | |  |  | | **2** | |
|  |  |  |  |  |  |  |  |  | **1** | 1 | 3 | **I** |  |  |  |  |  |  |  |  |  |  |  |
|  |  | **GS-2** |  |  |  |  | | **GS-2** |  |  |  |  | **GS-2** | |  | |  | |  |  | |  | |
|  |  | **GS-3** |  |  |  |  | | **GS-3** |  |  |  |  | **GS-3** | | **1** | | 2 | | 4 | **I** | | **1** | |

EGUS, Equine Gastric Ulcer syndrome; EGGD, Equine Glandular Gastric Disease; ESGD, Equine Squamous Gastric Disease; I, Improvement of the Total Severity Score in comparison to the first gastroscopic examination; L, developed lameness between first and second gastroscopic examination; *n*, number; No, no improvement in the Total Severity Score in comparison to the first gastroscopic examination; oD, other diseases; GS, Glandular Score; SS, Squamous Score; W, worsening of the Total Severity Score in comparison to the first gastroscopic examination
